# Spectral and raw *quasi in-situ* energy dispersive X-ray data captured via a TEM analysis of an ODS austenitic stainless steel sample under 1 MeV Kr^2+^ high temperature irradiation

**DOI:** 10.1016/j.dib.2017.08.030

**Published:** 2017-09-01

**Authors:** Adam J. Brooks, Zhongwen Yao

**Affiliations:** Queen's University, Reactor Materials Testing Laboratory, Kingston, Ontario, Canada

**Keywords:** Energy dispersive X-ray, Transmission electron microscope, *In-situ* irradiation

## Abstract

The data presented in this article is related to the research experiment, titled: ‘*Quasi in-situ* energy dispersive X-ray spectroscopy observation of matrix and solute interactions on Y-Ti-O oxide particles in an austenitic stainless steel under 1 MeV Kr^2+^ high temperature irradiation’ (Brooks et al., 2017) [1]. *Quasi in-situ* analysis during 1 MeV Kr^2+^ 520 °C irradiation allowed the same microstructural area to be observed using a transmission electron microscope (TEM), on an oxide dispersion strengthened (ODS) austenitic stainless steel sample. The data presented contains two sets of energy dispersive X-ray spectroscopy (EDX) data collected before and after irradiation to 1.5 displacements-per-atom (~1.25×10^−3^ dpa/s with 7.5×10^14^ ions cm^−2^). The vendor software used to process and output the data is the Bruker Esprit v1.9 suite. The data includes the spectral (counts vs. keV energy) of the *quasi in-situ* scanned region (512×512 pixels at 56k magnification), along with the EDX scanning parameters. The.raw files from the Bruker Esprit v1.9 output are additionally included along with the.rpl data information files. Furthermore included are the two *quasi in-situ* HAADF images for visual comparison of the regions before and after irradiation. This *in-situ* experiment is deemed ‘*quasi’* due to the thin foil irradiation taking place at an external TEM facility. We present this data for critical and/or extended analysis from the scientific community, with applications applying to: experimental data correlation, confirmation of results, and as computer based modeling inputs.

**Specifications Table**

**Table:** 

Subject area	Materials Science
More specific subject area	*Nuclear Materials*
Type of data	*Energy Dispersive X-Ray (EDX) spectral, and Bruker.raw*
How data was acquired	*FEI Tecnai Osiris*^*TM*^*Scanning Transmission Electron Microscopy*
Data format	*.txt (spectral) and.raw (Bruker EDX format)*
Experimental factors	*110* *nm TEM thin foil, 512×512 pixels at 56k magnification*
Experimental features	*EDX data from before and after irradiation*
Data source location	*Data collection from Queen's University Reactor Materials Testing Laboratory (RMTL): 136 Grant Timmins Dr, Kingston, ON K7M 8N3, Canada*
Data accessibility	*Files can be accessed in the Open Science Framework*[2]: www.osf.io/5ezcb
	*DOI:10.17605/OSF.IO/5EZCB*

**Value of the data**

• *In-situ* EDX microscopy data is useful for elemental analysis, and to see how the material X-ray energy output changes before and after experiment.
• Applicable for comparing elemental change vs. dose in both experimental and computational based models.
• Spectral *in-situ* data helps characterize any global changes of elements in the scanned region of material. The .raw data can be used, for example, with post processing in MATLAB, Python or ImageJ to slice and average the energy counts into a visible RGB 2D image spectra.

## 1 Data

This EDX data-in-brief (DiB) collection is from a 520 °C before and after irradiation experiment to ~1.5 displacements-per-atom (~1.25×10^−3^ dpa/s with 7.5×10^14^ ions cm^−2^) of the same microstructural area. The ChemiSTEM^TM^ EDX data was captured using a 200 keV FEI Tecnai Osiris in STEM mode. Ref. [2] has a link to the open source spectral and raw data files, as the raw files are 500 MB+ in size. They are available for download, through the Open Science Framework. This DiB also contains the *in-situ* high-angle annular dark-field imaging (HAADF) images for visual comparison of the regions before and after irradiation. The material scanned from EDX is a 310-ODS austenitic stainless steel modeled after commercial grade 310L (25Cr-20Ni-2Mo-0.02C-0.4N-0.35Y_2_O_3_-0.5Ti-bal.Fe), which is synthesized from a powder metallurgy fabrication route using hot isostatic pressing. Reference the corresponding article from Brooks et al. [1], to gain an understanding of the theory and experimental methods this data applies to.

## 2 Experimental design, materials and methods

### 2.1 Subsection 1: EDX hardware

The EDX mapping hardware has four windowless Super-X SDD detectors. Scanning line dwell time was set at 5 μs for a 512×512 pixel frame, with an average detector saturation dead time of 30–40%, depending on the area and magnification. The scans were held for 10 min, allotting sufficient time to collect a full spectrum. The software used to process the EDX data and output the .txt and .raw files is the Bruker Esprit suite v1.9. General processing settings for element identification and quantification is based off the *Φ*(ρz) (Phi-Rho-Z) with standards curves, which permits simultaneous absorption and matrix-based atomic number corrections to be made. This involves measuring the intensities of the X-ray lines in the unknown scanned region, pixel by pixel, and comparing those same lines to suitable standards, using identical instrumental conditions such as: accelerating voltage, beam current and beam size. Table 1 shows the EDX settings for the before irradiation data set. Each .txt file in [2] has the EDX settings for the before and after irradiation case.

**Table 1:** EDX hardware scanning information from the before irradiation data set. This chart is pulled from ‘Maximum pixel spectrum before.txt’ which can be found in [2].

Real time	38
Life time	38
Pulse density	713,263
Primary energy	200
Take off angle	22
Tilt angle	0
Azimut angle	45
Detector type	Custom type
Window type	Custom type
Detector thickness	0.45
Si dead layer	0.08
Calibration, lin.	10.002
Calibration, abs.	−944.689
Mn FWHM	136.832
Fano factor	0.118
Channels	4096

### 2.2 Subsection 2: Spectral figures and HADDF images

**Fig. 1:**
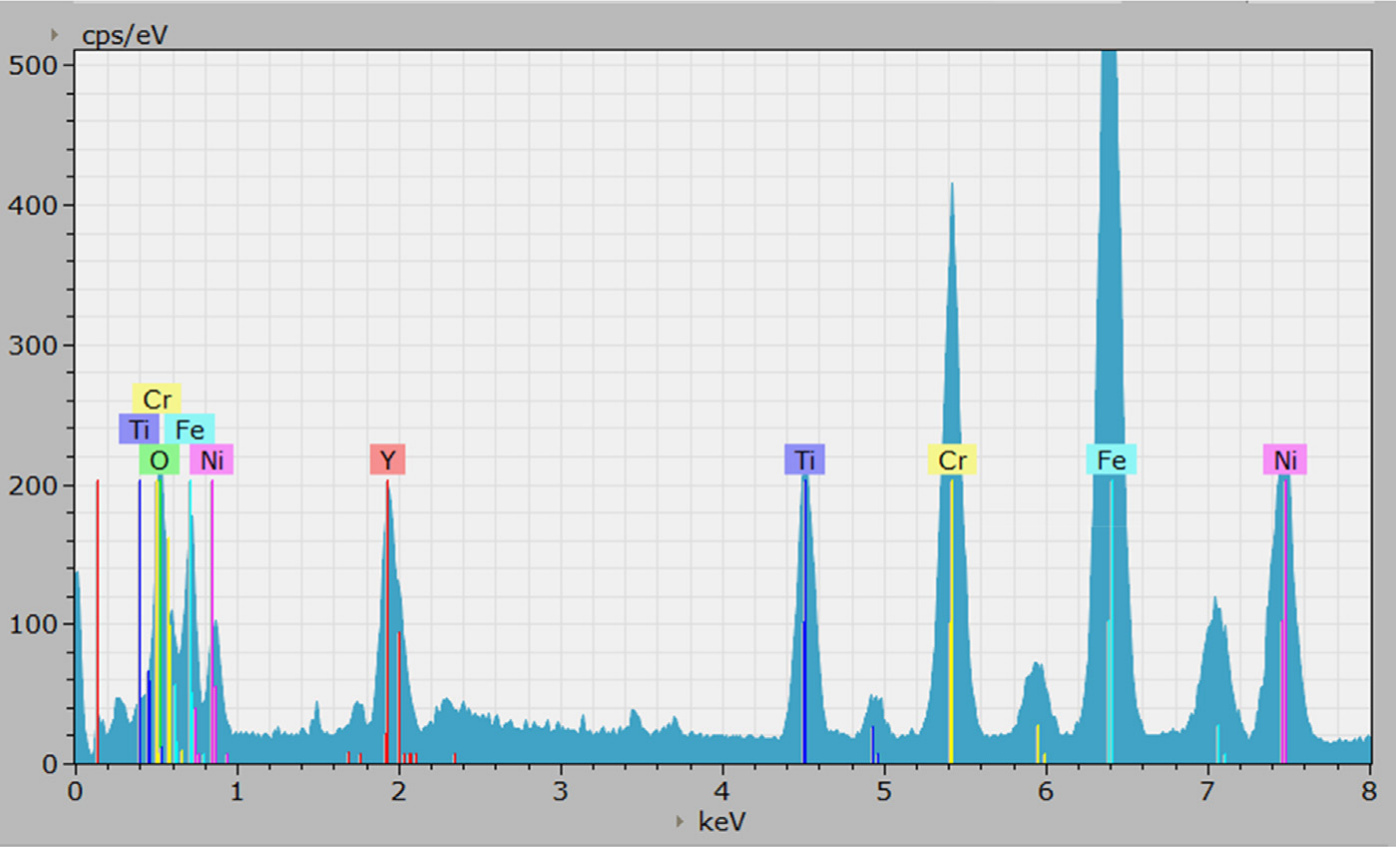
Counts vs. X-ray keV spectral chart for the before irradiation data, with highlighted elements from the 310-ODS material.

**Fig. 2:**
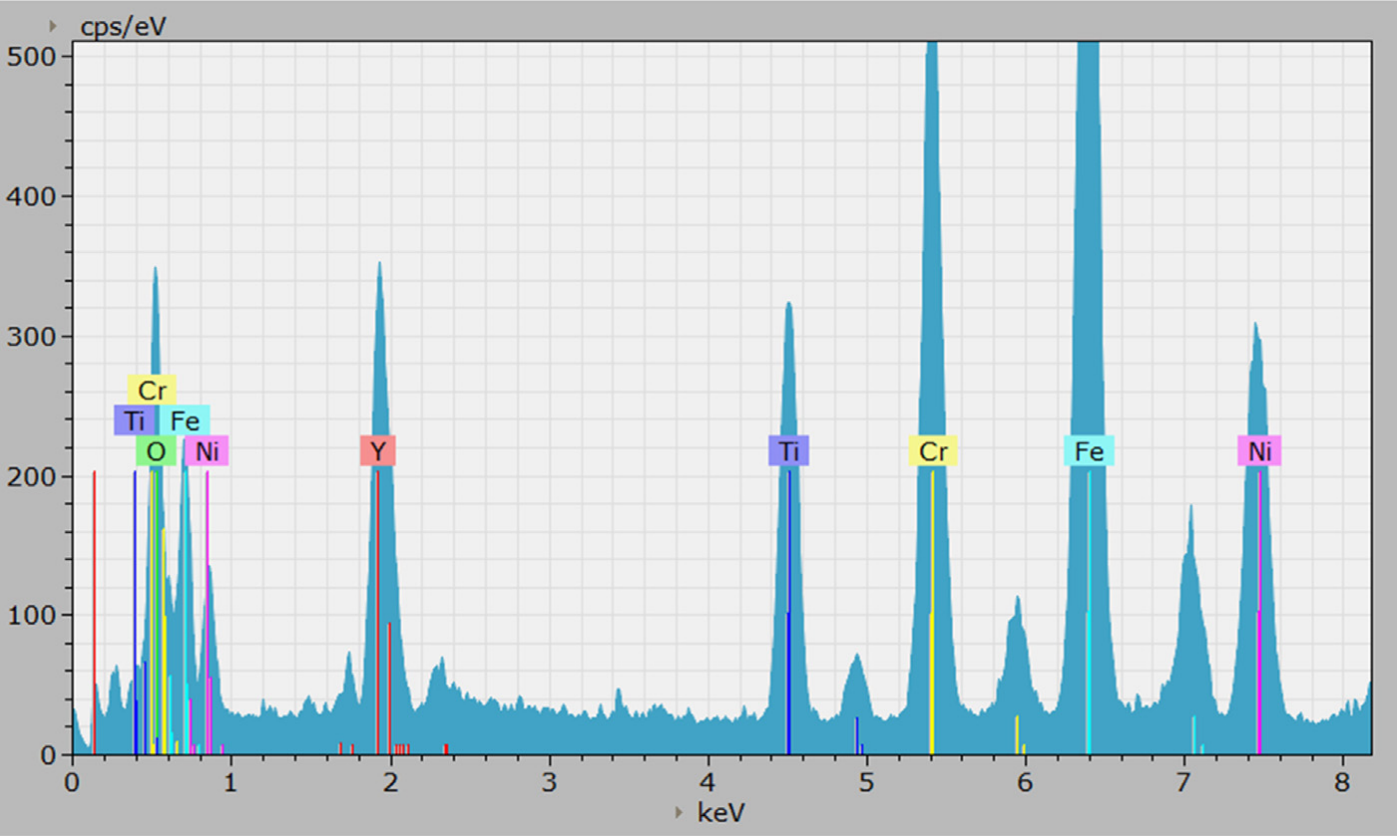
Counts vs. X-ray keV spectral chart for the after irradiation data, with highlighted elements. The spectra had more noise on the bottom end and higher global counts in the scanned region, due to the difference of 2048 channels vs. 4096 data channels in the before set. This channel difference was a result of user based data collection settings, bringing the workable data down from >1 GB (in the before case, Fig. 1) to 512 MB, which is easier to handle. The channel difference however does not influence the relative proportions of signal collection, it is simply binned into smaller packets, which results in greater displayed relative amplitudes.

**Fig. 3:**
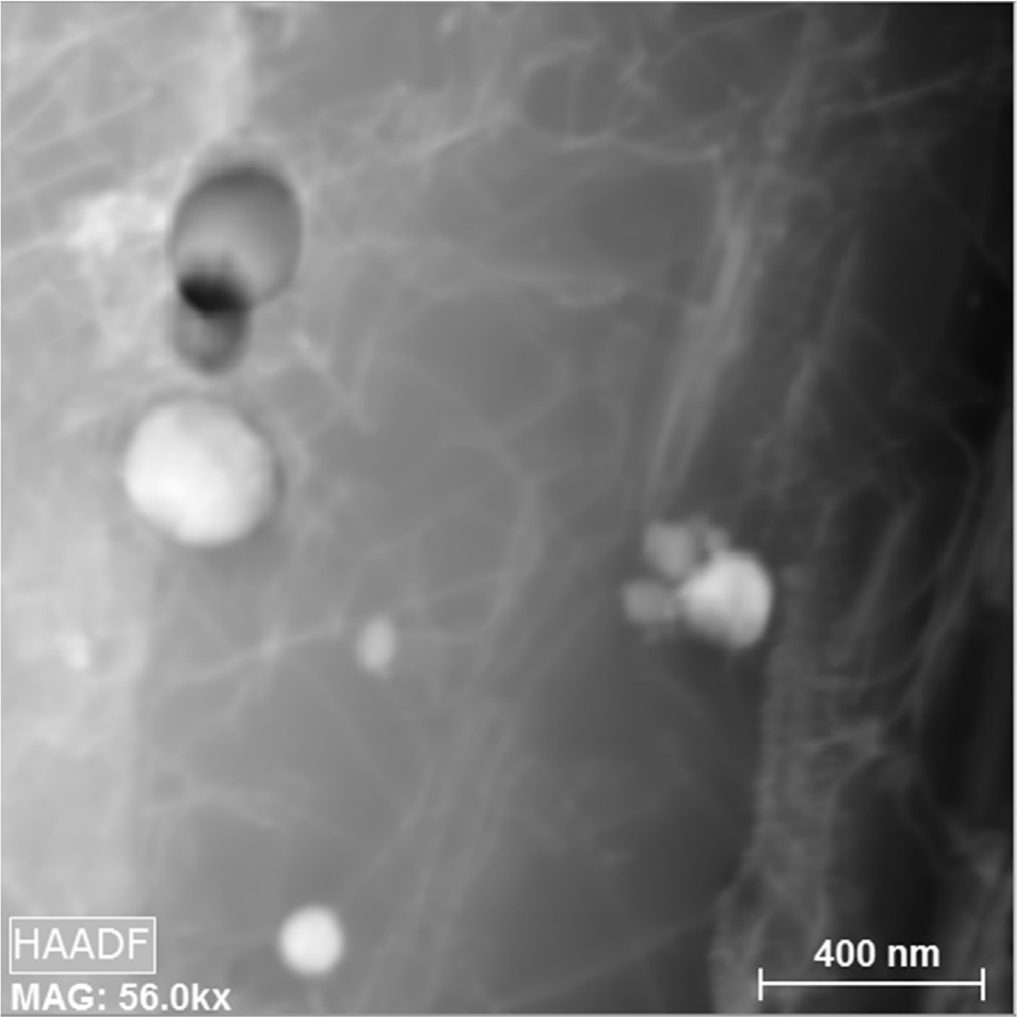
HAADF image of the (310-ODS) austenitic stainless steel before irradiation, 512×512 pixel region (~3.5 nm/pixel). The (white & black) contrast particles are Y-Ti-O oxides embedded within the Fe matrix.

**Fig. 4:**
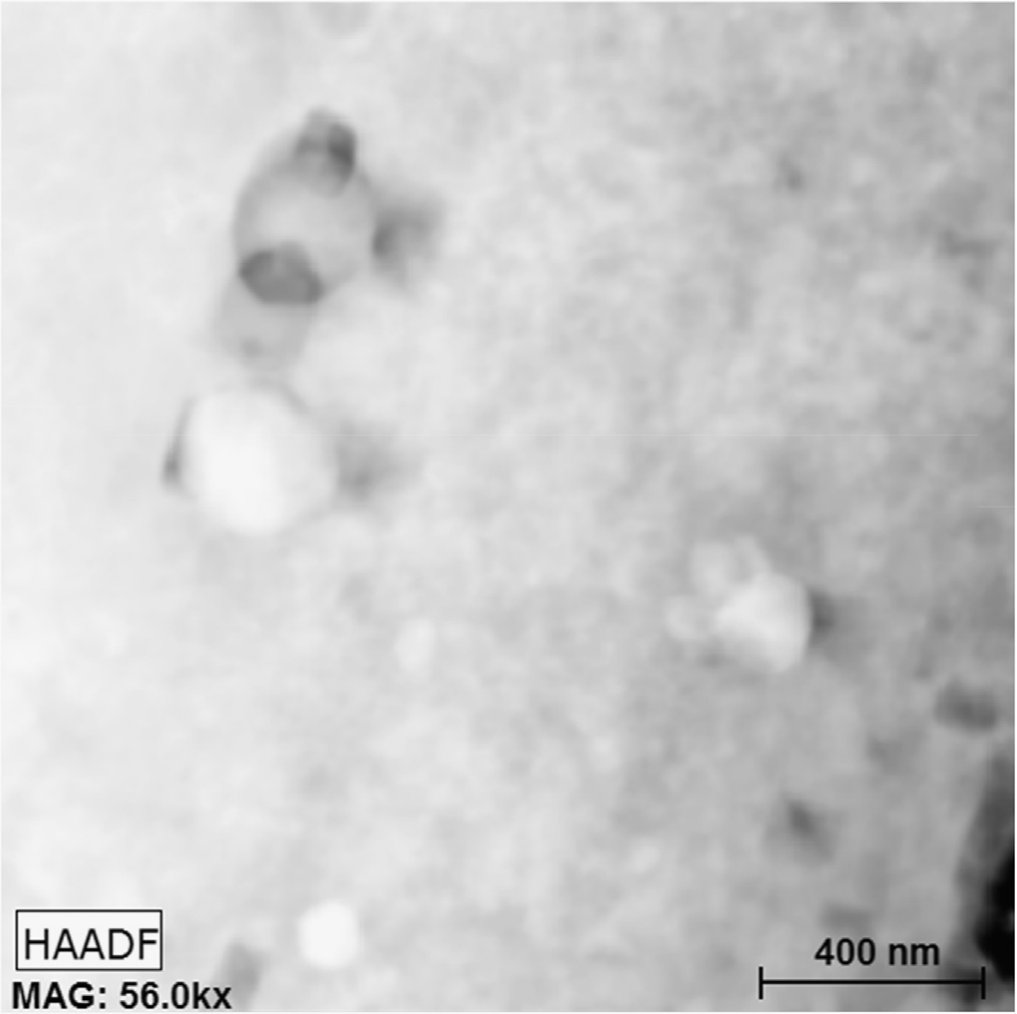
HAADF image of the (310-ODS) austenitic stainless steel after irradiation to 1.5 dpa (displacements-per-atom) at 520 °C, 512×512 pixel region (~3.5 nm/pixel).

The spectrum spatial resolution is based on the maximum pixel real distance, which varies in squared size from scan to scan depending on the magnification. In our case, the pixel real distance resolved to ~3.5 nm/pixel, at a magnification of 56,000×. Each pixel is then compiled into a counts/keV spectrum chart for the entire pixel area, and can be found in the .txt files [2]. Displayed in Fig. 1, Fig. 2 from the vendor software are the charts showing counts vs. X-ray energy captured (keV), which contains most of the useful information. Highlighted in the figures are the X-ray shell energy lines of the common elements found in the material: Fe, Ni, Cr, Y, Ti, O. Note, these spectrum charts are for the whole scanned area found in the HADDF images, Fig. 3, Fig. 4, and not from an individual section within the data set.

Furthermore, to accompany the spectral data and to help with visualization, included with this DiB are the high-angle annular dark-field (HAADF) images of the two regions. Fig. 3, Fig. 4 has the HAADF images before and after irradiation, respectfully. The contrast change after irradiation can be attributed to many surface dislocations, and possible deposited atmospheric surface contamination, a common artefact of high temperature thin foil irradiation. Ref. [1] goes into the detail of this contrast difference from experiment.

### 2.3 Subsection 3: Bruker.raw and.rpl Files

Lastly, the .raw files contain black and white contrast slices of each EDX energy channel (4096 for the before, and 2048 in the after scan) of the 512×512 pixel frame. Reference the files in [2]. The.rpl files are used as identifiers to the.raw data sets. An example.rpl from the before data set is listed in Table 2. The .raw files are useful in averaging the slices over a select energy range to produce 2D visible elemental maps, or to perform quantitative analysis of the X-ray energy channels. Subsequent .raw post processing, for example, can be done in MATLAB, Python or ImageJ.

**Table 2:** Example .rpl identification file from the before irradiation.raw set. This chart is pulled from ‘Before irr .rpl’ which can be found in [2]. The main indicators are the channel width and height, and the bit depth.

Key	Value
Width	512
Height	512
Depth	4096
Offset	0
Data-length	1
Data-type	Unsigned
Byte-order	Dont-care
Record-by	Vector
